# Multi-molecular ^14^C evidence for mineral control on terrestrial carbon storage and export

**DOI:** 10.1098/rsta.2022.0328

**Published:** 2023-11-27

**Authors:** Hannah Gies, Maarten Lupker, Valier Galy, Jordon Hemingway, Brenna Boehman, Melissa Schwab, Negar Haghipour, Timothy I. Eglinton

**Affiliations:** ^1^ Department of Earth Sciences, ETH Zürich, 8092 Zürich, Switzerland; ^2^ Woods Hole Oceanographic Institution, 360 Woods Hole Road, Falmouth, MA 02543, USA; ^3^ MIT-WHOI Joint Program in Oceanography/Applied Ocean Science and Engineering, Cambridge and Woods Hole, MA, USA; ^4^ Jet Propulsion Laboratory, California Institute of Technology, Pasadena, CA 91109, USA; ^5^ Department of Earth Sciences, ETH Zürich, 8093 Zürich, Switzerland

**Keywords:** organic carbon cycle, radiocarbon, biomarker, fluvial carbon, carbon turnover

## Abstract

Compound- and compound class-specific radiocarbon analysis of source-diagnostic ‘biomarker’ molecules has emerged as a powerful tool to gain insights into terrestrial carbon cycling. While most studies thus far have focused on higher plant biomarkers (i.e. plant leaf-wax *n*-alkanoic acids and *n*-alkanes, lignin-derived phenols), tracing paedogenic carbon is crucial given the pivotal role of soils in modulating ecosystem carbon turnover and organic carbon (OC) export. Here, we determine the radiocarbon (^14^C) ages of glycerol dialkyl glycerol tetraethers (GDGTs) in riverine sediments and compare them to those of higher plant biomarkers as well as markers of pyrogenic (fire-derived) carbon (benzene polycarboxylic acids, BPCAs) to assess their potential as tracers of soil turnover and export. GDGT Δ^14^C follows similar relationships with basin properties as vegetation-derived lignin phenols and leaf-wax *n*-alkanoic acids, suggesting that the radiocarbon ages of these compounds are significantly impacted by intermittent soil storage. Systematic radiocarbon age offsets are observable between the studied biomarkers, which are likely caused by different mobilization pathways and/or stabilization by mineral association.

This article is part of the Theo Murphy meeting issue 'Radiocarbon in the Anthropocene'.

## 1. Introduction

The continental biosphere is a major carbon reservoir, and understanding terrestrial ecosystem carbon dynamics is crucial to predict future responses of this carbon pool to climate change. Compound- and compound class-specific radiocarbon (^14^C) analysis has emerged as a powerful tool to gain insights into the dynamics of carbon cycling by tracing the fate of source-diagnostic ‘biomarker’ molecules, including the production of terrestrial biomass, fluvial export and deposition in aquatic sediments [1]. Thus far, research has primarily focused on two classes of higher plant biomarkers: leaf wax lipids (e.g. long-chain *n*-alkanoic ‘fatty’ acids and *n*-alkanes) and lignin-derived phenols [2]. Frequent observations of ^14^C-depleted radiocarbon signatures in the former class implies significant pre-ageing in intermediate reservoirs [3–6]. Terrestrial ecosystem turnover is largely modulated by soils, which constitute the largest continental carbon pool [7], and evidence suggests that the majority of terrestrial biospheric carbon exported by rivers is derived from erosion of mineral soils (e.g. [8,9]). Current models increasingly place microbially mediated processes as key drivers of soil carbon turnover and stabilization (e.g. [10,11]), but we currently lack diagnostic markers that reflect microbial processes and that serve as tracers of soil inputs to downstream carbon reservoirs [12]. Radiocarbon signatures of organic molecules that reflect the functioning and export of the soil organic matter pool would thus provide crucial constraints on carbon cycling in the environment.

Glycerol dialkyl glycerol tetraethers (GDGTs) are microbial membrane lipids that are found ubiquitously in aqueous and terrestrial environments [13]. GDGTs are subdivided into two groups of compounds: isoprenoid GDGTs (isoGDGTs) are produced by Archaea [14], while branched GDGTs (brGDGTs) are of bacterial origin [15–17]. BrGDGTs are especially abundant in soils and peats [15] and have thus been proposed as tracers of soil-derived OC [18] although aquatic brGDGT production and thus overprinting of the soil-derived signal has also been suggested (e.g. [19–21]). GDGTs have been a focus of extensive research due to their potential as molecular proxies to reconstruct paleoenvironmental conditions (e.g. [22–27]). Constraining the lateral transport and riverine *in situ* production of isoGDGTs and brGDGTs based on compound class-specific radiocarbon analysis is thus not only beneficial for the understanding of soil carbon turnover and erosional export, but also for the application of these molecules as paleoenvironmental proxies.

In this study, we determine the radiocarbon age of isoGDGTs and brGDGTs in riverine sediments from catchments located in different climatic and environmental settings. We analyse the impact of basin properties on GDGT radiocarbon age and compare the ^14^C signatures of these compounds to those of other biomarkers of terrestrial biospheric carbon measured on the same samples. We find that GDGT Δ^14^C values suggest a predominant origin from mineral-stabilized soil components. Systematic ^14^C offsets between the studied compounds reflect different organic matter storage and transport vectors.

## 2. Methods

### (a) Radiocarbon age of glycerol dialkyl glycerol tetraethers

Radiocarbon data of fatty acids, lignin, *n*-alkanes and BPCAs have previously been reported and is collected from the literature. Samples for GDGT analysis were chosen based on size (several hundred grams of riverine sediment are typically needed to obtain sufficient quantities of GDGTs for ^14^C measurement) and on the availability of reference data of other biomarkers, either for the same sample or close within the same catchment. Eight sediment samples were sieved to less than 63 µm and prepared for GDGT radiocarbon analysis: riverbed sediment from the Amazon [28,29], Gaoping and Kagayan [9] rivers; suspended sediment from the Koshi [30], Pearl, Earn, Dul [9]; and Arctic Red [31] rivers; and a floodplain deposit from the Danube River [32].

Extraction and radiocarbon analysis of GDGTs follows the procedure described in Gies *et al*. [33]. Briefly, the samples were extracted using an Energized Dispersive Guided Extraction (CEM EDGE) system in batches of 15–20 g of material. Lipids were extracted using 25 ml dichloromethane (DCM) : methanol (MeOH) 9 : 1 (v/v). Extraction temperature was set to 110°C for 2 min. followed by rinsing with 15 ml and a subsequent extraction with 5 ml of solvent at 100°C and rinsing with 35 ml. Extracts were pooled and dried under nitrogen (N_2_) flow, then 5 ml of MilliQ water with NaCl were added. The neutral phase was back-extracted with hexane (Hex) and separated on a 1% deactivated silica column into apolar and polar fractions with Hex : DCM 9 : 1 (v/v) and DCM:MeOH 1 : 1 (v/v), respectively. Polar fractions were dried under N_2_, then re-dissolved in Hex : 2-propanol (IPA) 99 : 1 (v/v) and passed over 0.45 µm PTFE filters immediately prior to high performance liquid chromatography (HPLC). Separation of isoGDGTs and brGDGTs is achieved on an Agilent 1260 HPLC system using two Waters Acquity UHPLC HEB hydrophilic liquid interaction chromatography (HILIC) columns (1.7 µm; 2.1 × 150 mm) connected in series coupled to an Agilent 1260 fraction collector. The columns are heated to 45°C and the flow rate set to 0.2 ml min^−1^. For the first 25 min, compounds elute isocratically with a solvent mixture of 18% Hex : IPA 9 : 1 (v/v; solvent A) and 82% hexane. The proportion of solvent B is then linearly reduced to 6% in 15 min, followed by a linear gradient to 0% hexane in 20 min. The total runtime of one injection hence adds up to 60 min, followed by 20 min reequilibration with 82% hexane [33]. Due to their low concentration, GDGTs are isolated as classes of compounds rather than at a compound-specific level, with windows for fraction collection determined based on previous LC–MS analysis. Isolated GDGT fractions were tested for purity by re-analysis on the HPLC–MS before being dried and transferred into 0.025 ml tin capsules (Elementar 03951620) that were then measured using an elemental analyser coupled to a gas-ion-source equipped accelerator mass spectrometer (EA-AMS) [34] at the laboratory of Ion Beam Physics at ETH Zürich [35,36]. As the re-analysis of the sample does not reveal contamination not detectable by HPLC–MS including solvent residue and column bleed, the GDGT fractions of two reference samples, a Swiss surface soil (Δ^14^C = −65.7‰) and a lignite sample (Δ^14^C = −940.4‰), were prepared and measured to estimate contamination introduced in the course of sample preparation. The analysis of these reference samples indicates an average contamination of 2.62  ±  0.79 µg C with Δ^14^C = −413.4 ± 178.8‰; measured GDGT fractions were corrected for this contamination [33].

### (b) Compound-specific radiocarbon data of other compounds

Data on the other biomarkers is sourced from the literature. BPCA data are taken from Coppola *et al*. [29]; long-chain fatty acids and lignin data are taken from Eglinton *et al*. [9] and references therein; and *n*-alkane data are obtained from Kusch *et al*. [37], Gustafsson *et al*. [38], Tao *et al*. [8] and Feng *et al*. [39].

### (c) Statistical analyses of catchment properties and compound-specific radiocarbon

Catchment characteristics used for comparison with biomarker data are taken from Eglinton *et al*. [9]. They encompass a suite of (i) geomorphic variables, including the proportion of floodplain (% floodplain) and the average elevation and slope of the catchment; (ii) climatic factors including mean annual temperature (MAT), mean annual precipitation (MAP) and the seasonal coefficient of variability of temperature and precipitation and (iii) other environmental control variables, namely the proportion of the catchment impacted by anthropogenic activity, net primary productivity (NPP), soil organic carbon (OC) content and ^14^C age of topsoil (0–30 cm) and soil (0–100 cm), as well as soil and ecosystem turnover times (*τ*_soil_, *τ*_ecosystem_). All basin properties influence the stability, erosion and mobilization of OC (e.g. [12,40–44]). Relations between catchment properties and compound or fraction dated are explored using a spearman rank-order correlation [45]. The sample size varies depending on the compound (bulk C *n* = 43, lignin *n* = 16, long-chained fatty acids *n* = 44, br and isoGDGTs *n* = 8, *n*-alkanes *n* = 13, BPCAs *n* = 19).

## 3. Results

**Figure 1.**
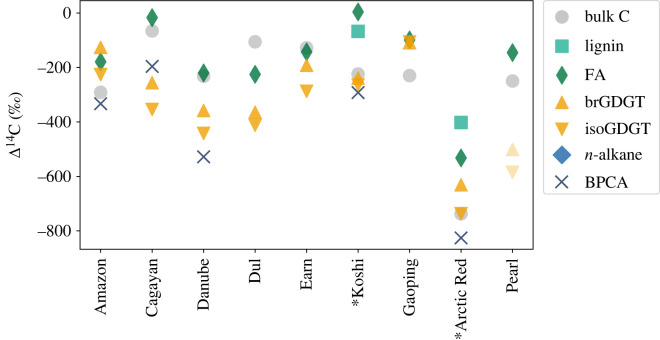
Δ^14^C values of brGDGTs and isoGDGTs in comparison to other specific compounds from the same or nearby river sediment samples. The asterisk (*) indicates that GDGTs were extracted from a different sample within the same major catchment than the other biomarkers. Specifically, GDGT data from the Koshi River are compared to biomarker data from the Ganges River, whereas GDGT data from the Arctic Red River are compared to data from the mainstem Mackenzie River. FA = long-chain fatty acids.

**Figure 2.**
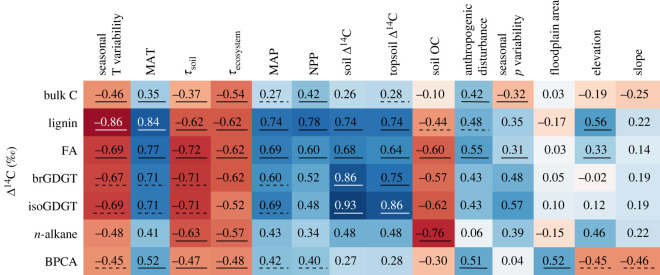
Spearman correlation coefficients for biomarker/bulk OC radiocarbon age and basin properties. Each cell shows the Spearman correlation coefficient for the Δ^14^C values of the respective compound class and environmental parameter. Solid lines indicate a *p*-value < 0.05, a dashed line corresponds to a *p*-value < 0.1. If the value is not underlined, the *p*-value of the correlation is greater than 0.1. Colours represent the sign (blue—positive correlation, red—negative correlation) and the strength of the correlation, with a darker shade corresponding to a higher absolute correlation coefficient. FA = long-chain fatty acids.

Measured radiocarbon content of GDGTs in riverine sediments are shown in figure 1 and electronic supplementary material, table S1. Δ^14^C values of isoGDGTs and brGDGTs are similar to one another in all samples: the largest difference between the two GDGT groups (−106‰) is observed in the Arctic Red River. In all samples apart from the Gaoping sediment, isoGDGTs are more depleted in Δ^14^C than corresponding brGDGTs. Δ^14^C values of isoGDGTs and brGDGTs range between −106‰ and −737‰ and −109‰ and −631‰, respectively. Except for the Amazon and Gaoping sediments, most samples feature GDGTs with lower Δ^14^C values (older radiocarbon ages) than corresponding plant-derived biomarkers lignin and long-chain fatty acids as well as bulk POC and soil carbon. In the Amazon, Koshi, Danube and Arctic Red rivers, GDGT Δ^14^C values are higher than BPCA values. The Pearl River features low Δ^14^C values (brGDGTs: −501‰, isoGDGTs: −585‰) in comparison to bulk OC (−249.6‰) and fatty acids (−145.3‰). Due to the large offset compared to the other samples, the Pearl River GDGT data is not included in the statistical analysis (figure 2). As Δ^14^C values of isoGDGTs and brGDGTs are similar in each sediment sample, the correlations with catchment properties are similar for both compound classes. With the exception of floodplain area, elevation and slope, most correlation coefficients are close to or exceed 0.5. Nevertheless, only the correlations with soil Δ^14^C and topsoil Δ^14^C are significant (*p*-value < 0.05) due to the small sample size of 8 (excluding the Pearl River).

Long-chain fatty acids and lignin phenol Δ^14^C values correlate similarly with basin properties in most cases, except for the stronger correlation of soil carbon content with fatty acid Δ^14^C compared to that of lignin, and the stronger correlation of elevation with lignin phenol Δ^14^C compared to that of fatty acids. Δ^14^C values of both biomarker groups are significantly influenced by the selected catchment properties, with the exception of floodplain area and mean slope. The correlations of long-chain fatty acids and lignin Δ^14^C values are similar to both groups of GDGTs in strength and direction.

The radiocarbon content of long-chain *n*-alkanes is significantly correlated with soil OC, *τ*_ecosystem_ and *τ*_soil_, while the correlations with the other catchment properties are weaker compared to those of long-chain FAs, lignin phenols and GDGTs (coefficients typically *r*_s_ < 0.5). Similarly, the response of BPCA Δ^14^C values to catchment properties is weaker compared to those of other biomarkers, with coefficients *r*_s_ ranging between 0.04 and 0.52. Nevertheless, only BPCAs are significantly correlated with floodplain area and slope. Finally, bulk OC Δ^14^C correlations with catchment variables are in general weaker compared to specific classes of biomarker compounds.

**Figure 3.**
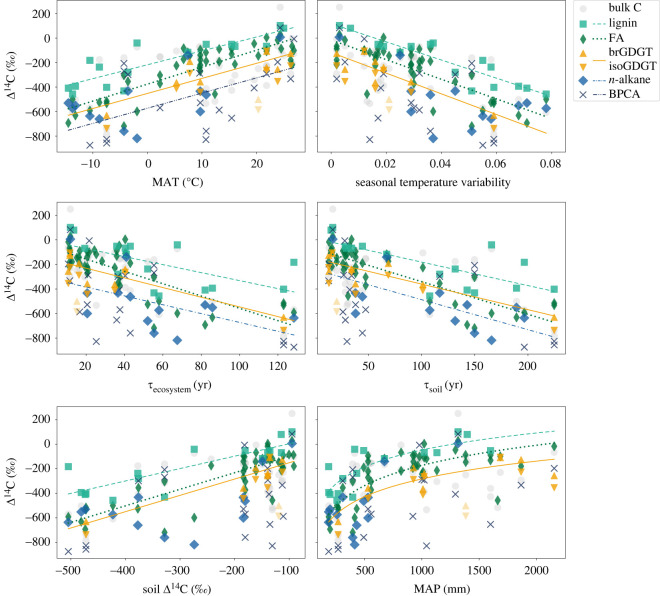
Δ^14^C values of bulk OC and biomarkers plotted against different basin properties. The displayed properties were selected as they explain most of the variability of biomarker Δ^14^C values. Fitted functions are shown for GDGTs and for other biomarkers that feature a correlation coefficient *r*_s_ > 0.5 with the respective catchment parameter. The transparent brGDGT and isoGDGT symbols shows the Δ^14^C values of the Pearl river, which is considered an outlier. Uncertainties (1*σ*) are smaller than symbols.

The six basin properties that explain most of the variability in biomarker Δ^14^C values are displayed in figure 3. Overall, the best fits for functions between each biomarker and the respective catchment property indicate that for a given value of a catchment property, Δ^14^C values for specific biomarkers exhibit the following trend, from higher (younger) to lower (older): lignin—long-chain fatty acids—long-chain *n*-alkanes—GDGTs—BPCAs (see also electronic supplementary material, figure S2).

## 4. Discussion

BrGDGTs are especially abundant in soils and peats [15], hence brGDGTs were proposed as a tracer of soil OC input into marine and lacustrine sediments (e.g. [18,46,47]). More recently, this assumption has been challenged by observations of *in situ* brGDGT production in lakes and coastal marine environments (e.g. [48–51]) as well as in rivers [19–21,52]. Specifically, brGDGT distributions in riverine sediments have been observed to diverge from those in surrounding soils, implying some *in situ* production of these compounds. As all of the rivers analysed for GDGTs in this study are quite turbid due to high suspended sediment loads, a pre-requisite to accrue the needed amount of sediment for GDGT ^14^C analysis, we assume low *in situ* production. The low Δ^14^C values of both isoGDGTs and brGDGTs also support this presumption: isoGDGTs are thought to be produced by Thaumarchaeota [13], chemoautotrophic nitrifiers that fix bicarbonate [53,54]. IsoGDGTs produced in rivers should reflect the radiocarbon age of dissolved inorganic carbon (DIC). However, reported DIC radiocarbon, where available, is less depleted in ^14^C than the isoGDGTs in the river sediment (Amazon: 120‰ [55], Cagayan: −17‰ [56], Pearl River: −90‰ [57], Koshi: −206‰ [58]). Similarly, the source organisms of brGDGTs, though not yet identified unequivocally [16,17], are supposedly heterotrophic bacteria [15,59,60]. Except for the Amazon and the Gaoping sample, brGDGTs are more depleted in ^14^C than bulk OC, and, by extension dissolved OC, which is generally younger than the particulate carbon fraction [61]. Neither the radiocarbon signatures of isoGDGTs nor of brGDGTs can be sufficiently explained with *in situ* production. We, therefore, conclude that the majority of these compounds originate from soils in the respective river catchments.

In all catchments except for the Gaoping and the Amazon (for brGDGTs), average soil Δ^14^C values between 0 and 100 cm are higher than GDGT Δ^14^C values in the sediment. Surficial erosion preferentially mobilizes topsoil for riverine export [43,62,63], which is usually more modern (i.e. higher Δ^14^C) than the overall soil profile. While current observations are limited, the radiocarbon age of GDGTs in topsoil is close to that of bulk soil OC [33], and thus GDGTs originally mobilized and introduced to the river via surficial pathways are presumably younger than the GDGTs that are measured in the fluvial sediments. Organic matter degrades on a range of time-scales, even on the compound-class level [64]. In soils, GDGTs show turnover times in the range of millennia, indicating the presence of a slow-cycling GDGT pool that likely is protected from degradation due to association with mineral surfaces [33]. Upon mobilization, younger labile GDGTs might be preferentially degraded en route, while an older, mineral-stabilized pool persists, manifesting itself in the sediments. The preferential degradation of freshly produced marine GDGTs compared to stabilized continental GDGTs has been observed in turbidites [65], the same process might already occur during fluvial transport, as evidenced by the low Δ^14^C values of riverine GDGTs.

Eglinton *et al*. [9] analysed radiocarbon age global patterns for long-chained fatty acids as well as lignin phenols in riverine sediments, and they showed a link between OC turnover in soils and the age of these biomarkers. The correlations of GDGT Δ^14^C values with different basin properties are in many cases similar in strength compared to those for the long-chain fatty acids and lignin phenols (figures 2 and 3). Given that GDGTs in the studied rivers are predominantly sourced from soils within the catchment, the low Δ^14^C values indicate that a significant fraction of GDGTs is pre-aged and represents a soil-derived mineral-stabilized OC pool. Correlations between GDGT Δ^14^C and environmental parameters indicate the turnover of this mineral-stabilized fraction is correlated with the respective parameter. Long-chain FAs and lignin phenols are vegetation derived; however, the correlations of their Δ^14^C values with catchment characteristics resemble those of soil microbial GDGTs, implying that despite their different origins, turnover of all three biomarker classes is largely driven by mineral stabilization, confirming the observations of Eglinton *et al*. [9].

There are at least two non-exclusive potential explanations for the systematic offsets in Δ^14^C values between the compound classes (figures 1 and 3), with lignin phenols generally being least and GDGTs most ^14^C-depleted under the same catchment and environmental conditions. First, this could reflect different sizes of fast (mineral-free) and slow (mineral-associated) cycling pools of these compounds, potentially as a result of different mobilization pathways (surface runoff versus deeper mineral soil erosion). Alternatively, these compounds may exhibit varying degrees of mineral association [66]. For example, lignin is generally a minor component of mineral-stabilized soil organic matter [67], whereas long-chain fatty acids are preferentially associated with the mineral fraction of soils [3,68] and GDGTs with the fine grain size fraction of sediments [69]. In either case, the relative size or degree of mineral protection could explain the observed age differences for GDGTs and long-chain fatty acids compared to lignin. Further studies are needed to fully elucidate underlying causes.

Similar to long-chain FAs, long-chain odd-carbon-numbered *n*-alkanes are constituents of cuticular leaf waxes [2]. However, while their age is also significantly correlated to soil properties in the catchment (soil %OC, *τ*_ecosystem_ and *τ*_soil_), climatic factors such as MAP, MAT and seasonal temperature differences appear to more weakly influence *n*-alkane Δ^14^C compared to the corresponding FAs (figure 2). Moreover, *n*-alkane Δ^14^C values are lower (older) than those of fatty acids from the same fluvial systems. While fatty acids are entirely sourced from vegetation, *n*-alkanes in sediments can also be sourced from sedimentary bedrock [2], or derive from anthropogenic (fossil fuel) contamination (e.g. [70]). The addition of fossil hydrocarbons from either source may suppress the ages of *n*-alkanes and weaken the relationship with climatic variables. The correlations of BPCA Δ^14^C values with catchment properties do not resemble the patterns of the other biomarkers examined here, implying that processes governing turnover, mobilization and transport of pyrogenic carbon differ markedly from those that control the export of most biospheric carbon.

The transport and deposition of biospheric carbon is an integral part of the global carbon cycle on both short and long timescales. Our results highlight that mineral stabilization in soils is a dominant control on whether biospheric carbon is respired in transit or buried in sediments for both vegetation- and microbially derived organic material. Furthermore, all biomarkers included here are used as paleoenvironmental proxies (e.g. [2,13,71–73]). The systematic age offsets observed in riverine samples indicate that biomarkers co-accumulating in lacustrine sediments or shelf deposits have experienced different predepositional histories (soil turnover, mobilization pathways) and may be differently pre-aged prior to translocation and deposition, and hence may not reflect the same environmental conditions [6].

## 5. Conclusion

We have analysed the radiocarbon ages of microbially derived isoGDGT and brGDGT fractions in riverine sediments and compared them to other source-specific biomarkers to assess their potential as tracer of soil OC dynamics in river basins. We find that in the studied river samples, GDGT Δ^14^C values suggest a predominant origin from mineral-stabilized soil components. The similar correlations of GDGT, lignin and long-chain fatty acid Δ^14^C with environmental parameters imply that the latter vegetation-derived compounds must also emanate from a mineral-stabilized pool in soils that experiences pre-ageing prior to mobilization and export. Systematic ^14^C age differences between these components reflect either differing mobilization of different proportions of mineral-poor (younger) and mineral-associated (older) biomarkers, or differing degrees of mineral association of the different molecular components. Contrasting ^14^C characteristics of long-chain *n*-alkane and BPCAs must reflect different sources or transformation/transport pathways. Our results highlight the potential of molecular-level radiocarbon measurements to elucidate the dynamics of specific carbon pools and to track the fate of these pools from source to sink. Our findings also underline the need for in-depth investigation of the processes involved in mineral stabilization of organic matter and their impact on continental OC dynamics under environmental change, as well as on sedimentary molecular proxy records.

## Data Availability

The data are provided in electronic supplementary material [74].
